# Experience of multi-disciplinary treatment of multiple cerebellar diffuse large B-cell lymphoma: A case report

**DOI:** 10.1097/MD.0000000000037923

**Published:** 2024-04-26

**Authors:** Yongji Guo, Juntao Li, Mengda Li, Zhixiao Li, Yongxia Cui, Yan Li, Ruijiao Zhao, Qian Han, Hongqi Yang, Chunxiao Ma

**Affiliations:** a Department of Neurosurgery, People’s Hospital of Henan University, Zhengzhou, China; b Department of Neurosurgery, Henan Provincial People’s Hospital, Zhengzhou, China; c Department of Oncology, Henan Provincial People’s Hospital, Zhengzhou, China; d Department of Imaging, Henan Provincial People’s Hospital, Zhengzhou, China; e Department of Pathology, Henan Provincial People’s Hospital, Zhengzhou, China; f Department of Radiotherapy, Henan Provincial People’s Hospital, Zhengzhou, China; g Department of Neurology, Henan Provincial People’s Hospital, Zhengzhou, China.

**Keywords:** case report, cerebellar tumor, chemotherapy, diffuse large B-cell lymphoma, radiotherapy

## Abstract

**Rationale:**

Primary central nervous system lymphoma (PCNSL) is a rare, highly malignant form of non-Hodgkin lymphoma categorized under the diffuse large B-cell type. It accounts for merely 1% of all non-Hodgkin lymphoma cases and comprises approximately 3% of all brain tumors. The involvement of the cerebellum is observed in only 9% of these cases. Recently, we came across an unusual instance: a young man presenting with multiple lesions located specifically within the cerebellum.

**Patient concerns:**

A 26-year-old male was admitted to the hospital due to severe headaches. He has a medical history of sporadic headaches, accompanied by dizziness, nausea, and vomiting persisting for a month. Over the last 10 days, his headaches have intensified, coupled with decreased vision and protrusion of the eyeballs. Magnetic resonance imaging (MRI) revealed abnormal signals in both cerebellar hemispheres.

**Diagnoses, interventions, and outcomes:**

Diagnostic procedures included cerebellar biopsy, posterior fossa decompression, and lateral ventricle drainage. Histopathological examination identified diffuse large B-cell lymphoma (DLBCL) with high proliferative activity. To minimize neurotoxicity, chemotherapy involved intrathecal methotrexate (MTX) injections combined with the CHOP program. The patient has shown good tolerance to the treatment so far.

**Lessons:**

While the definitive optimal treatment approach remains elusive, current chemotherapy centered on high-dose MTX stands as the standard induction therapy. Integrating surgery with radiotherapy and chemotherapy significantly extends patient survival.

## 1. Introduction primary

Primary central nervous system lymphoma (PCNSL) is an uncommon and highly malignant form of non-Hodgkin lymphoma, characterized as diffuse large B-cell lymphoma (DLBCL) specifically confined to the central nervous system (CNS).^[1]^ It involves areas such as the brain, spinal cord, brain nerves, eyes, and meninges, representing merely 1% of all non-Hodgkin lymphoma cases and accounting for around 3% of all brain tumors.^[2,3]^ In recent decades, its incidence has shown an upward trend, primarily affecting older individuals with compromised immunity, while occurrences among young individuals with intact immune function are rare. PCNSL typically infiltrates the cerebral hemispheres, thalamus/basal ganglia, corpus callosum, and periventricular regions, with rare instances involving the cerebellum.^[4]^ We encountered an unusual case featuring multiple PCNSL lesions within the cerebellum of a young individual with a normal immune system, marking a rare presentation of this condition.

## 2. Patient information

**Figure 1.**
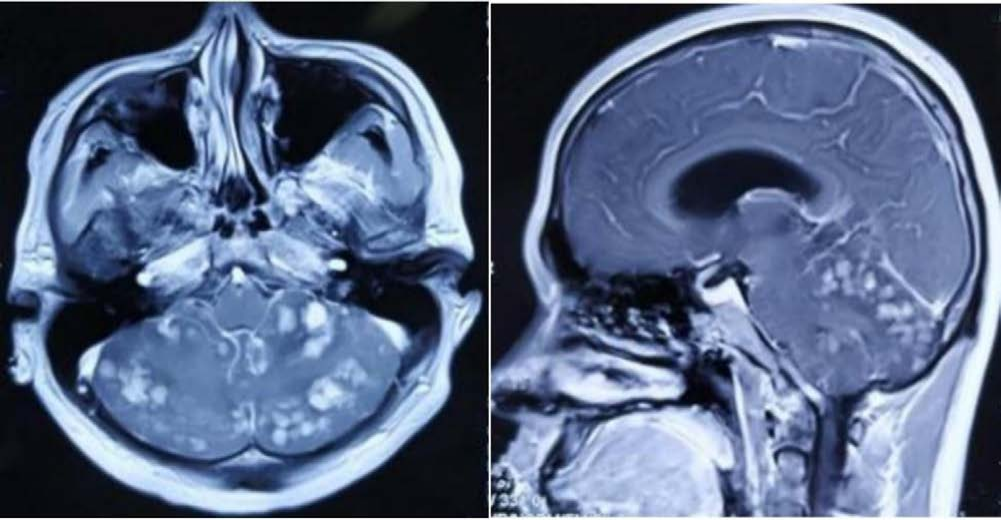
The enhanced MRI showed multiple abnormal signals in the vermis cerebellum and bilateral cerebellar hemispheres. MRI = magnetic resonance imaging.

A 26-year-old man presented at our hospital emergency department complaining of persistent headache, dizziness, and blurred vision for the past 10 days. Upon admission, an enhanced magnetic resonance imaging (MRI) of the head revealed multiple abnormal signals in the right temporal lobe, cerebellar vermis, and bilateral cerebellar hemispheres, with some involvement of the meninges. Additionally, there was a reduction in the size of the fourth ventricle (refer to Fig. 1). Initial investigations via ultrasound and X-ray failed to indicate any primary tumors outside the CNS. The pattern of these cerebellar lesions is highly suggestive of metastatic disease; however, the absence of a discernible primary lesion contradicts the typical features associated with cerebellar metastasis. In addition, the disease was not found in the patient family members.

**Figure 2.**
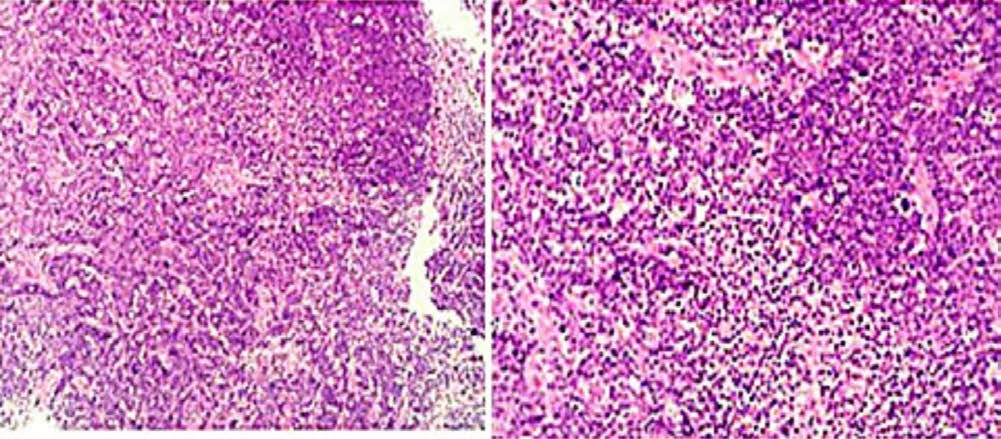
The histopathological sections revealed extensive diffuse infiltration of B lymphocytes, indicating heightened activity.

**Figure 3.**
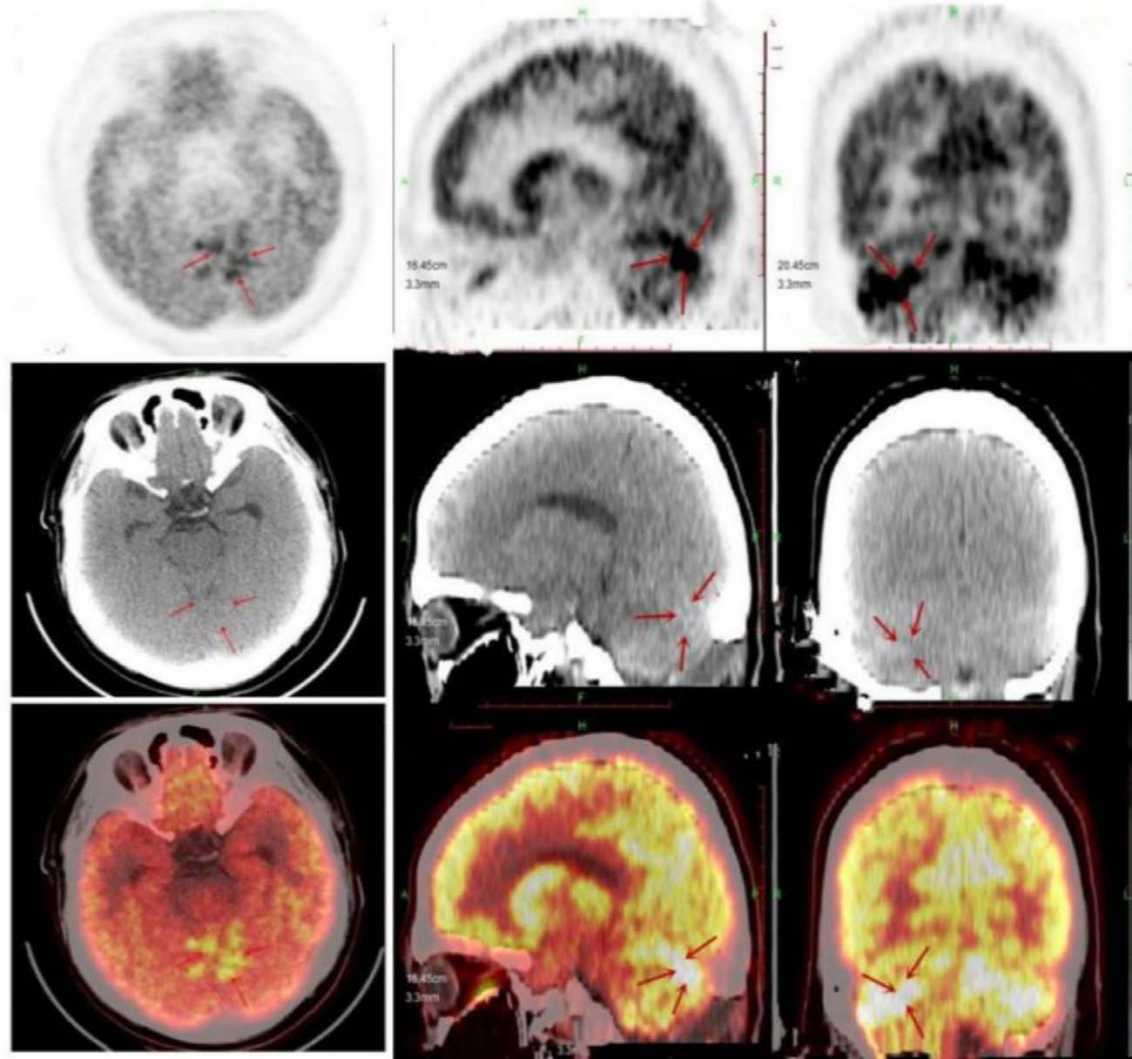
The PET scan revealed patchy mixed density in the cerebellum, displaying inhomogeneous radioactivity uptake and heightened local metabolism.

As the patient symptoms progressively worsened, our diagnostic considerations encompassed lymphoma, intracranial infection (specifically tuberculosis), or metastasis. To both diagnose the condition and alleviate the escalating clinical symptoms, a lumbar puncture was performed, revealing an intracranial pressure elevation of up to 400 mmH2O. Cerebrospinal fluid (CSF) analysis demonstrated glucose levels of 0.01 mmol/L, CSF protein levels of 1.06 g/L, and normal results for hemogram, TB spot test, tuberculosis, expert-resistant tuberculosis, and acid-fast staining. Subsequent second-generation sequencing and exfoliated cell examination revealed no discernible abnormalities. However, these comprehensive tests failed to provide a definitive diagnosis. Due to the critical escalation of intracranial pressure, necessitating urgent intervention, the medical team decided upon cerebellar biopsy, posterior fossa decompression, and peripheral examination. Histopathological analysis leaned toward non-Hodgkin lymphoma, specifically DLBCL, exhibiting CD20 and CD10 positivity, a Ki67 proliferation index exceeding 80%, a germinal center type, and high-value-added activity (refer to Fig. 2). postoperation, a whole-body positron emission tomography-computed tomography (PET-CT) scan illustrated patchy mixed density shadows within the cerebellum, displaying unevenly increased radioactive uptake. Notably, no primary tumors were detected outside the CNS (refer to Fig. 3).

Further investigations, including bone marrow biopsy and testicular ultrasound, revealed no evidence of lymphoma in these specific areas. Additionally, the test for human immunodeficiency virus returned negative results. To minimize neurotoxicity, a chemotherapy regimen was initiated, involving methotrexate (MTX) intrathecal injections combined with the CHOP program. As of the latest assessment, the patient has exhibited excellent tolerance to the treatment. Subsequent enhanced computed tomography (CT) scans have shown no residual lesions. posttreatment, the patient symptoms have notably improved, with significant enhancements in visual acuity and a reduction in headache symptoms.

## 3. Discussion

PCNSL is an infrequent yet aggressive form of DLBCL. Its incidence typically rises with age, often manifesting around a median age of 65 years.^[5]^ While human immunodeficiency virus/acquired immune deficiency syndrome remains a significant risk factor,^[6]^ recent studies highlight a growing occurrence in older individuals with normal immune function.^[2]^ The disease progression of PCNSL tends to be swift, lasting approximately 6 months, with 5- and 10-year survival rates averaging 29.9% and 22.2%.^[7]^ Its primary symptoms involve space-occupying effects or indications of intracranial hypertension, such as headaches and vomiting caused by diffuse brain edema. Cognitive impairment and altered consciousness are also prevalent.^[8]^ Focal signs, including seizures, papilledema, paralysis, aphasia, and dysarthria, correlate with the tumor location and extent.^[9]^ Elevated CSF protein levels are commonly observed, with tumor cells detected in roughly 50% of patients, often presenting increased lymphocyte proportions. Electroencephalograms for about 80% of patients exhibit abnormalities, displaying focal or diffuse lesions. However, these clinical manifestations lack specificity, leading to potential misdiagnosis during the early stages, often mistaken for inflammation, resulting in anti-inflammatory treatments.^[10]^ PCNSL typically presents as solitary or multiple nodular lesions in the brain, necessitating differentiation from meningioma, glioma, intracranial metastasis, or inflammation. Imaging studies indicate varying densities on CT and MRI, typically characterized by isointensity or low intensity on T1-weighted images and isointensity or high intensity on T2-weighted images. The tumor boundary is usually well-defined, with minimal occurrences of cystic changes, necrosis, or calcification.^[11]^ Once diagnosed, prompt determination of lymphoma expansion and immediate implementation of effective treatment measures are imperative. The standard treatment for PCNSL primarily relies on high-dose methotrexate multi-chemotherapy, often followed by whole-brain radiotherapy.^[7]^ Surgery primarily serves for stereotactic biopsy to establish a histopathological diagnosis. While surgery alone does not significantly improve survival rates or quality of life, some studies suggest its potential survival advantages, especially when followed by postoperative adjuvant radiotherapy.^[12]^ Treatment for PCNSL predominantly revolves around radiotherapy and chemotherapy. Despite the high sensitivity of PCNSL to these treatments and significant symptom relief posttreatment, recurrence rates remain high, limiting overall efficacy.^[13]^ For relapsed or refractory cases, high-dose chemotherapy with autologous stem cell transplantation represents the most advanced treatment option, showcasing the best outcomes.^[14]^ However, due to limited research, no standard treatment regimen exists for PCNSL. Retrospective studies indicate that radiotherapy and chemotherapy extend average survival times. Nevertheless, disagreements persist regarding the sequence and approach to chemotherapy and radiotherapy, whether to prioritize chemotherapy or radiotherapy initially, or opt for single chemotherapy or combined chemotherapy-radiotherapy regimens. In contrast, for elderly patients with compromised health, initiating treatment with chemotherapy might be preferable to reduce neurotoxicity from chemical radiation. Nonetheless, it might still result in cognitive dysfunction posttreatment.^[13]^

In this case, treatment involved MTX intrathecal injections combined with the CHOP regimen (cyclophosphamide + adriamycin + vincristine). Some studies have suggested that high-dose methotrexate + rituximab + whole-brain radiotherapy combination therapy for PCNSL yields improved efficacy and prognosis^[15]^; however, further prospective research is warranted to validate these findings. Sierra et al^[16]^ conducted comparative studies, treating 39 out of 69 PCNSL patients with intrathecal methotrexate and 30 without. Their findings indicated no significant difference between the 2 groups (*P* > .05). While rituximab is a common drug for peripheral lymphoma treatment, its limited passage through the blood–CSF barrier restricts its use in PCNSL. Recent studies have indicated that rituximab, when administered early in the disease course, can extend the survival period. Particularly in elderly patients with PCNSL, where tumors may compromise the blood–brain barrier, rituximab showcases remarkable effectiveness.^[17]^ Kraemer et al^[18]^ highlighted that intra-arterial chemotherapy, coupled with disrupting the permeable blood–CSF barrier, can enhance drug concentration to the tumor by 2 to 5 times, significantly surpassing concentrations achievable with intravenous chemotherapy alone. Thus, it evident that disrupting the blood–CSF barrier through alternative methods can notably augment drug concentrations in the CNS, leading to enhanced therapeutic effects.

According to several studies, patients who underwent radiotherapy before chemotherapy exhibited significantly longer survival periods compared to those who received chemotherapy before radiotherapy.^[19]^ The rationale behind this observation might stem from radiotherapy ability to disrupt the blood–CSF barrier, potentially facilitating enhanced drug penetration into the target area postradiotherapy. However, these studies face limitations, including fewer comparative cases and differing conditions among these cases, leading to uncertainties regarding the superiority of radiotherapy over chemotherapy. Despite potential benefits, the combination of chemotherapy and radiotherapy markedly increased neurotoxicity in most patients, resulting in unstable progression and diminished cognitive abilities. MRI scans revealed white matter atrophy and diffusion disorders, with higher risks of neurotoxicity among older patients. Hence, if chemotherapy alone can sufficiently address the issue, avoiding radiotherapy might be preferable. Currently, ibrutinib and linadolamine show promising potential in PCNSL treatment. However, relying solely on a new drug might not be feasible. Effectively combining these drugs with other chemotherapy regimens could yield unexpected therapeutic benefits.^[5]^

## 4. Conclusion

PCNSL, a rare tumor with a bleak prognosis, warrants a deeper understanding of its molecular genetic characteristics and prognostic indicators. Recent strides in PCNSL treatment have shown progress, yet recurrences remain frequent, and the prognosis remains unfavorable. While the definitive optimal treatment approach remains elusive, current chemotherapy centered on high-dose MTX stands as the standard induction therapy. Integrating surgery with radiotherapy and chemotherapy significantly extends patient survival, positioning it as a first-line treatment. However, due to PCNSL predilection for deep brain parenchyma, obtaining biopsies becomes challenging, emphasizing the criticality of early imaging-based diagnosis. Employing a combination of imaging modalities (CT, MRI, PET-CT, PET-MRI) alongside CSF cytology exams substantially enhances diagnostic accuracy. Presently, T1-weighted enhanced MRI serves as the standard imaging method, although definitive diagnosis still relies on pathological examination and immunohistochemistry. Timely diagnosis and intervention are pivotal for improving PCNSL prognosis. Researchers exploring PCNSL co-diagnostic markers have identified potential drivers like CXCL13, cytokines such as IL-10, which, when combined with imaging, aid in diagnosis. Additionally, microRNAs (21, 19b, 92a) detectable in CSF offer diagnostic support. However, these studies remain in the preclinical phase and necessitate validation through large-scale clinical trials. Despite the absence of a standardized treatment and the generally poor prognosis, recent years have witnessed a spectrum of novel treatment strategies that notably extend the average survival period for PCNSL patients posttreatment. I remain optimistic that continued human efforts will eventually triumph over this disease.

## Author contributions

**Conceptualization:** Chunxiao Ma.

**Investigation:** Mengda Li.

**Project administration:** Zhixiao Li.

**Resources:** Yongxia Cui, Yan Li, Ruijiao Zhao.

**Validation:** Qian Han, Hongqi Yang.

**Writing – original draft:** Yongji Guo, Juntao Li.

**Writing – review & editing:** Juntao Li, Chunxiao Ma.
